# Multiple Regulatory Signals and Components in the Modulation of Bicarbonate Transporters

**DOI:** 10.3390/pharmaceutics16010078

**Published:** 2024-01-05

**Authors:** Hyeong Jae Kim, Jeong Hee Hong

**Affiliations:** Department of Physiology, Lee Gil Ya Cancer and Diabetes Institute, College of Medicine, Gachon University, 155 Getbeolro, Yeonsu-gu, Incheon 21999, Republic of Korea; lilili1125@naver.com

**Keywords:** bicarbonate transporter, cellular homeostasis, channelopathy, inflammation, host defense mechanism

## Abstract

Bicarbonate transporters are responsible for the appropriate flux of bicarbonate across the plasma membrane to perform various fundamental cellular functions. The functions of bicarbonate transporters, including pH regulation, cell migration, and inflammation, are highlighted in various cellular systems, encompassing their participation in both physiological and pathological processes. In this review, we focused on recently identified modulatory signaling components that regulate the expression and activity of bicarbonate transporters. Moreover, we addressed recent advances in our understanding of cooperative systems of bicarbonate transporters and channelopathies. This current review aims to provide a new, in-depth understanding of numerous human diseases associated with the dysfunction of bicarbonate transporters.

## 1. Homeostatic Role of Bicarbonate Transporters

Bicarbonate is a fundamental ion in cellular pH regulation and crosses the membrane via various bicarbonate transporters, such as the chloride/bicarbonate exchangers (CBE; SLC26A family and anion exchanger (AE) family), sodium/bicarbonate cotransporter (NBC), sodium-dependent chloride–bicarbonate exchanger (NDCBE), and the cystic fibrosis (CF) transmembrane conductance regulator (CFTR) channel [1,2,3,4,5]. It has been known that the human adult bicarbonate level is maintained between 23 to 29 mEq/L in serum and 10 mEq/L in the cytosol [6]. Since bicarbonate transporters were first identified, their biological and physiological roles have been revealed in various cellular systems, including pathological processes. Defects in bicarbonate transporters result in disease-state signaling [7]. Although acid-base cellular pH regulation and basic physiological concepts of bicarbonate transporters are highlighted in various reviews [8,9,10,11], the regulatory signals or components that act on the modulation of bicarbonate transporters are relatively unknown. This review summarizes recent advances in signaling components and the bicarbonate-associated enzymes in the modulation of bicarbonate transporter function and the understanding of bicarbonate transporters to develop drug targets against bicarbonate transporter-associated diseases.

## 2. Modulatory Signals in the Regulation of Bicarbonate Transporters

### 2.1. Inflammatory Disease-Associated Cytokines

Inflammatory cytokines increase bicarbonate secretion in epithelia [12,13]. The pH of airway epithelial surface liquid, for example, is a critical factor in respiratory defense against inhaled pathogens [14]. Alkalization of the cell surface fluid through bicarbonate secretion is associated with host defense mechanisms. Thus, the roles of bicarbonate transporters in inflammatory settings have been investigated in several studies of airway and intestinal systems, and these findings are discussed in this section.

Among the chloride–bicarbonate exchangers, downregulated in adenoma (DRA, also termed SLC26A3) is found in the gastrointestinal system and is mainly implicated in inflammatory diseases. For instance, the interleukin (IL)-10-deleted colitis mouse model and patients with ulcerative colitis revealed reduced DRA expression [15]. The downregulation of DRA leads to a defect in bicarbonate secretion and may affect the protective function against overt diarrhea [16]. 

In addition, inflammatory diseases, such as bowel disease, reveal the enhanced tumor necrosis factor (TNF)-α levels in the mucosa and lamina propria [17,18]. Several studies have verified the relationship between bicarbonate transporters and TNF-α. Treatment with TNF-α reduces mRNA and protein expression of DRA in colorectal adenocarcinoma HT-29 cells and Caco-2 3D-cysts model [19]. Moreover, injection of TNF-α decreases mRNA and protein expression levels of DRA compared to controls in crypt-derived mouse ileal enteroids on matrigel [19]. Overexpression of nuclear factor-kappa B (NF-κB) subunits also inhibits mRNA and protein expression levels of DRA [19]. Conversely, mRNA expression of DRA is increased by the treatment of NF-κB inhibitor caffeic acid phenethyl ester in HT-29 cells and ileal enteroids [19]. In ileal enteroid-derived mouse crypts, TNF-α decreases DRA mRNA expression, while having no effect on another CBE, *Slc26a6* (mouse PAT-1) [19]. DRA knockout mice, not PAT-1 knockout, reveal dysregulated electrolyte balance; thus DRA, not PAT-1, is important in the regulation of intestinal chloride and fluid volume [20]. In ulcerative colitis (UC) patient samples, DRA expression levels are decreased, whereas serum TNF-α levels are upregulated compared to a control group [21]. Consistent with these findings, in a dextran sulfate sodium (DSS)-induced colitis mouse model, expression levels of DRA mRNA and protein are downregulated, whereas TNF-α is upregulated [21]. In DRA-overexpressed Caco2-brush border of enterocyte (Caco2/BBE) cells, TNF-α treatment induces the downregulation of DRA [21]. Knockdown of TNF-α with siRNA significantly upregulates mRNA and protein expression of DRA [21]. Conversely, knockdown of DRA with siRNA induces the upregulated mRNA and protein expression of TNF-α compared to control [21]. Thus, stability of DRA might be affected by inflammatory cytokines and DRA activity is dominantly involved in the maintenance of electrolyte homeostasis. 

Additionally, it is known that anion exchanger 2 (AE2) secretes bicarbonate to protect biliary mucosa in the digestive system from related diseases such as primary biliary cholangitis (PBC) [22]. Pro-inflammatory cytokines such as IL-8, IL-12, IL-17, IL-18, and TNF-α may contribute to the reduced expression and activity of AE2 by enhancing miR-506 expression in H69 cholangiocytes [23]. Dysregulated AE2 may impair the bicarbonate umbrella against bile salt-associated cellular apoptosis [23,24].

In addition to DRA and AE2, pendrin, also termed SLC26A4, has been associated with the airway system. IL-17 treatment time-dependently enhances mRNA and protein expression of pendrin in human bronchial epithelial (HBE) cells [25]. DRA mRNA is decreased with IL-17 treatment in HBE cells [25]. Regarding DRA expression in tissues, DRA is not detected in the respiratory system [26]. In the presence of the NF-κB inhibitor JSH-23, IL-17 treatment does not increase pendrin mRNA and protein expression in HBE cells [25]. In cystic fibrosis (CF) HBE cells, pendrin mRNA expression and pH_i_ are increased, whereas treatment with siRNA-pendrin decreases chloride/bicarbonate exchanger activity [25].

The combined administration of TNF-α and IL-17 time-dependently elevates the pH of the airway epithelial surface liquid [14]. Theoretically, the pH of airway epithelial liquid may increase as a consequence of increased bicarbonate secretion, decreased H^+^ secretion, or both [14]. These results indicate that TNF-α acidifies the pH of airway epithelial liquid by enhancement of H^+^ secretion, whereas IL-17 has no effect on H^+^ transport. Additionally, co-treatment with both TNF-α and IL-17 increases the pH of airway epithelial liquid through enhanced bicarbonate secretion, not by reduced H^+^ secretion, in human airway epithelia [14].

**Figure 1:**
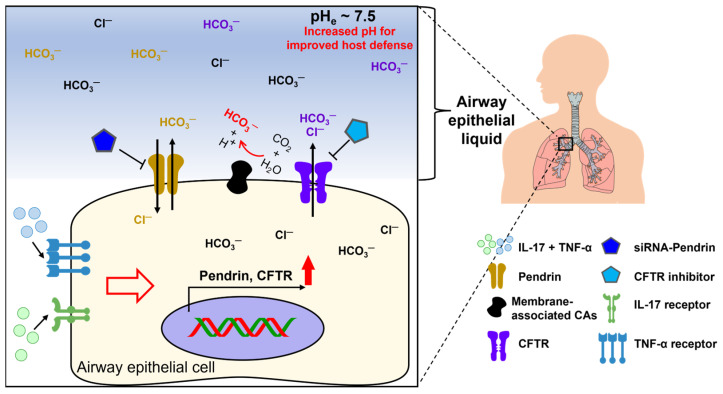
Schematic illustration of inflammatory cytokine-mediated ion transporters in airway epithelia. Bicarbonate secretion by airway epithelial transporters plays a crucial role in the barrier defense mechanism against inflammation by enhancing the pH of airway epithelial fluid. The presence of TNF and IL-17 in airway epithelial fluid enhances the efficacy of CFTR and pendrin function [29]. CFTR: cystic fibrosis transmembrane conductance regulator, CAs: carbonic anhydrases (enhanced membrane-associated CA9 and CA12) [14].

The combined treatment of IL-17 and TNF-α increases the mRNA expression of a gene subset of bicarbonate transporters, including CFTR, members of the SLC26A family, and the SLC4 family (NBCs) and its associated enzymes, carbonic anhydrases (CAs), in human airway epithelia [14]. We included CFTR in this review because the chloride channel CFTR is considered as the bicarbonate efflux transporter [27,28]. Concomitant addition of IL-17 and TNF-α with CFTR (inh)-172, a CFTR inhibitor, induces decreased pH of airway epithelial liquid [29]. Co-treatment of IL-17 and TNF-α with CFTR modulators (VX-445, VX-661, and VX-770) causes an increase in the pH of airway epithelial surface liquid [29]. Additionally, treatment with pendrin siRNA in the co-presence of IL-17 and TNF-α induces reduced pH of airway epithelial liquid [14]. Thus, combined treatment with IL-17 and TNF-α induces bicarbonate secretion through the involvement of CFTR and pendrin [29], alkalinizing the pH of airway epithelial liquid and providing an important barrier defense mechanism against airway inflammation (Figure 1). Understanding the effects of cytokines on the pH regulation of airway surface fluid through bicarbonate transporters would support the development of therapeutic strategies against airway inflammation.

### 2.2. Angiotensin II-Mediated Modulation of Bicarbonate Transport

Angiotensin II (ANG II) regulates systemic and renal circulation through the release of aldosterone in the adrenal cortex. It is known that ANG II is involved in bicarbonate reabsorption in the proximal tubule [30]. ANG II-mediated bicarbonate absorption occurs through the involvement of the bicarbonate transporter NBC in proximal tubules [30,31,32]. Wang et al. [33] demonstrated that the addition of ANG II increases fluid and bicarbonate absorption rates in the early distal tubules. However, ANG II fails to stimulate bicarbonate absorption in the late distal tubules. In an in vivo study of separated tubule segments, treatment with ANG II increased fluid absorption, while its antagonist Sarilesin decreased fluid absorption in both early and late distal tubules [33]. Thus, they found that ANG II stimulates bicarbonate and Na^+^ reabsorption in the proximal tubule and showed that ANG II receptors are located in both the lumen and basement membrane of proximal tubules [33]. In addition to ANG II-mediated bicarbonate absorption by NBC, ANG II stimulates sodium–hydrogen exchanger (NHE) activity in proximal and early distal tubules and cardiac muscles [30,33,34]. Although we highlighted the effect of ANG II on NBC and NHE in the renal system, the fundamental mechanism of acid-base transport and bicarbonate transporters in body system is described in several reviews [35,36]. 

ANG II receptors are categorized into two major subtypes: type 1 (AT1) and type 2 (AT2) receptors. ANG II-mediated calcium responses in the renal system are investigated using AT1 and AT2 antagonists, valsartan, and PD123319, respectively [37]. ANG II-mediated calcium responses are inhibited by treatment with valsartan but not PD123319 in isolated proximal tubules of wild-type (WT) mice. These results suggest that ANG II-evoked calcium responses are mediated by AT1 [37]. Additionally, isolated tubules of type A-AT1-knockout (KO) mice lose biphasic regulation of ANG II-mediated NBC activity [37]. ANG II stimulates NBC activity in adult ventricular myocytes from rats [38] and cats [39]. The hypertensive rat heart revealed impaired electrogenic NBC1 (NBCe1) activity and electroneutral type of NBC (NBCn1)-dependent pH recovery, suggesting that impaired NBCe1 activity by ANG II may be compensated by upregulated expression of NBCn1 in ventricular myocytes [40]. ANG II also mediates the production of reactive oxygen species (ROS), which activates NBC activity through the extracellular signal-regulated kinase (ERK) pathway in cat ventricular myocytes [39]. The ERK inhibitor U0126 treatment inhibits ANG II-induced equivalent acid efflux, indicating pH recovery through the involvement of NBC [39]. Furthermore, ANG II treatment inhibits the activity of NBCe1 through p38 kinase, not through an ERK1/2 pathway in cat cardiomyocytes [41]. Additionally, ANG II downregulates NBCe1 activation via p38-kinase activation, whereas NBCn1 activity is upregulated by ERK1/2- and ROS-dependent pathways in cat ventricular myocytes [40,41]. ANG II-activated AT1 receptors mediate increased calcium, alteration of the redox balance of cardiomyocytes, and subsequent cardiac hypertrophy through the activation of phospholipase C [42]. 

ANG II-mediated intracellular pH modulation is also involved in AE activity. ANG II treatment facilitates the reduction of myocardial intracellular pH by activating AE under conditions of an alkali load induced by trimethylamine hydrochloride (TMA), a protein kinase C (PKC) activator, treatment in cat papillary muscles [43]. Among the AE family members AE1 to AE3, full-length AE3 is involved in ANG II-mediated activation of PKC in the myocardium [44]. Additionally, the protein expression of AE1 is upregulated by a combination of the aldosterone analogue deoxycorticosterone acetate and NaHCO_3_ to induce alkalosis in the mouse kidney medulla, helping to maintain acid–base balance [45]. 

**Figure 2:**
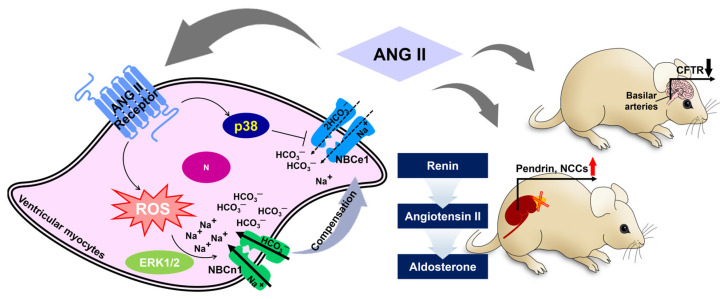
Schematic illustration of angiotensin II-mediated bicarbonate transporter regulation. ANG II represents differential roles in NBCs in ventricular myocytes [40,41]. There are differential effects of ANG II on pendrin/NCC and CFTR in the kidney and basilar arteries of the brain, respectively [46,47]. Black and red arrow indicates down- or up-regulated protein expression, respectively. ANG II: angiotensin II, ERK1/2: extracellular signal-regulated kinase1/2, ROS: reactive oxygen species, N: nucleus, NCCs: sodium chloride cotransporter, CFTR: cystic fibrosis transmembrane conductance regulator.

In addition, although sodium-chloride cotransporter (NCC) is not included in the bicarbonate transporter family, the NCC in plasma membrane is enhanced by ANG II treatment, and co-stimulation of aldosterone enhances pendrin expression, along with the expression of phosphorylated NCC, in adrenalectomized mice [46]. This implies that the upregulation of NCCs and pendrin by ANG II is required for the maintenance of blood pressure via the renin–ANG–aldosterone system. Furthermore, treatment with ANG II decreases CFTR mRNA and protein expression in the basilar arteries of the brain [47]. We illustrated the differential role of ANG II in Figure 2. We emphasized that CFTR is understood to be a regulator of vasoconstriction. Thus, the effect of ANG II on the modulation of bicarbonate transporters is complex and requires careful consideration, taking into account the different functions of bicarbonate transporters such as pendrin and CFTR in each organ.

### 2.3. Regulation of Bicarbonate Transport by Endogenous Peptides

#### 2.3.1. Neuropeptide Vasoactive Intestinal Peptide

Vasoactive intestinal polypeptide (VIP) is a 28-amino-acid neuropeptide produced by neurons, T cells, B cells, and endocrine cells and is found in most organs [48,49]. VIP is released in response to various stimuli, including serotonin, acetylcholine, substance P, and others [49]. VIP stimulation contributes to the regulation of inflammation and smooth muscle relaxation [50]. It also has been reported that VIP activates bicarbonate transport by involving CFTR in mice with disrupted CFTR genes, established as a colony model for cystic fibrosis [51]. 

VIP is a physiological activator of CFTR, contributing to mucus hydration and local nonspecific immune system activity in mammalian airways [52,53]. VIP enhances CFTR activity through both protein kinase A (PKA)- and PKC-dependent molecular mechanisms [54,55,56]. Stimulation of VIP receptors (VPAC1 and VPAC2) enhances cAMP and calcium production through the G protein-coupled VPACs and subsequently activates CFTR function [54,55,57]. Accordingly, VIP is a regulator of CFTR-mediated secretion in respiratory submucosal glands [52,58]. In Calu-3 lung epithelial cells, treatment with VIP time-dependently increases CFTR expression in the apical membrane [59]. VIP-induced CFTR expression is downregulated via the treatment of PKC inhibitors bisindolylmaleimide X or chelerythrine chloride [59]. 

Oxidative damage caused by ozone exposure or cigarette smoke leads to an upregulation of glutathione and a downregulation of chloride secretion by reducing CFTR expression levels at the apical side in Calu-3 and T84 cells [60,61]. Ozone stress induces the downregulation of CFTR expression and activity through the signal transducer and activator of transcription 1 (STAT1) signal pathway in HBE cells [62]. VIP treatment has been shown to recover ozone stress-mediated CFTR dysfunction in HBE cells through the PKA and PKC signaling pathway [56]. Furthermore, treatment with the PKCε inhibitor peptide, Epsilon-V1-2, reduces the surface expression of CFTR in the presence of VIP in Calu-3 cells [63], suggesting that PKCε is involved in VIP-modulated CFTR membrane stability. 

Sodium–hydrogen exchange regulatory factor-1 (NHERF1) is a PDZ domain-containing scaffolding protein that interacts with CFTR [64,65]. VIP exposure has been shown to enhance NHERF1 expression and co-localization with CFTR [63]. However, VIP treatment in the presence of siRNA-NHERF1 does not enhance the surface expression of CFTR compared to VIP treatment alone [63]. These results suggest that NHERF1 participates in the VIP-CFTR signaling cascade. Although VIP-mediated bicarbonate or chloride regulation is well-defined for CFTR function [66,67,68,69], the verification of regulatory interactions between VIP and other bicarbonate transporters should be investigated in the future.

#### 2.3.2. Neuropeptide Y

**Figure 3:**
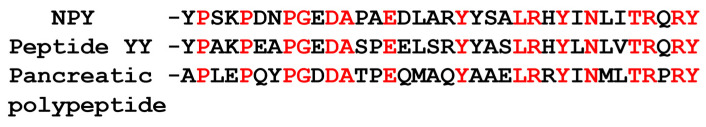
Amino acid sequence of NPY family members (NPY, peptide YY, and pancreatic polypeptide). The NPY family members share significant biological homology (red color) [75,76,77]. The multiple sequence alignment was performed with a CLUSTALW.

Neuropeptide Y (NPY) is a 36-amino acid peptide hormone extensively expressed in submucosal neurons and the myenteric plexus throughout the gastrointestinal tract of humans [70], mice [71], and rats [72]. The NPY family includes NPY, peptide YY, and pancreatic polypeptide [73] (sequences are illustrated in Figure 3), sharing significant biological homology [74,75,76,77]. Among them, NPY is known to be a vasoconstriction peptide [78]. Since NPY is secreted simultaneously with norepinephrine (NE) in sympathetic responses, NE-induced vasoconstriction is accelerated by NPY-mediated NPY Y1 receptor activation [78].

Sakena et al. investigated the short-term regulation of NPY on CBE to understand NPY-mediated chloride and sodium transport in the gastrointestinal tract [79]. NPY treatment upregulates CBE activity in Caco2 cells [79]. Furthermore, selective agonists for NPY/Y1 or Y2 receptors increase CBE activity, while the ERK1/2 inhibitor U0126 downregulates NPY-mediated CBE activity in Caco2 cells [79]. NPY treatment also enhances DRA expression in lipid raft fractions [79]. It is generally believed that the association of membrane transporters in lipid rafts is required for optimal activity of transporters [80], and cholesterol content is crucial for the integrity of lipid rafts [81]. Treatment with the cholesterol-sequestering agent methyl-β-cyclodextrin (MβCD) reduces DRA expression in Caco2 lipid rafts, and, conversely, reduced CBE activity is restored by cholesterol addition [79]. 

NPY and VIP exhibit opposing functions in CFTR-associated inflammatory reactions, where NPY stimulates cytokine release while VIP reduces cytokine secretion [67]. NPY treatment downregulates VIP-mediated acidification and cellular shrinkage through the inhibition of cAMP in primary serous cells [67]. The activation of cAMP is blocked by the VIP receptor antagonist VIP (6-28), and this effect is restored by VIP treatment [67]. These findings confirm that NPY treatment inhibits CFTR-mediated chloride, bicarbonate, and fluid secretion by reducing cAMP signaling. Consequently, elevated NPY levels may contribute to reduced fluid secretion and subsequently induce airway inflammation. Although it has been reported that NPY and VIP levels are increased in human nasal polyps, inflammatory cells of ovalbumin-induced chronic asthma mice, and inflammatory bowel disease (IBD) patients, both peptides are considered immune modulators in airway diseases [82,83,84] and their sophisticated regulation and disease-specific roles should be further investigated in the future.

### 2.4. Intracellular Calcium-Associated Modulation of Bicarbonate Transport

Intracellular calcium homeostasis is a critical process for maintaining various physiological functions, including cardiac and neural function [85,86], and is regulated by numerous signaling proteins in a highly coordinated manner. 

For instance, in a myocardial infarction mouse model, levels of NBCe1 mRNA and protein are upregulated in ventricular myocytes [87]. In cardiac-specific transgenic mice overexpressing human NBCe1, the mortality rate and infarct size are higher, and left ventricular (LV) systolic function and contractility are decreased compared to WT mice, suggesting that overexpression of NBCe1 results in dysfunction of the LV [87]. NBCe1 overexpression increases hypoxia-induced sodium and calcium signaling changes in adult mouse ventricular myocytes [87]. However, treatment with the NBC inhibitor S0859 reduces the concentration of these two ions in the setting of NBCe1 overexpression and hypoxia [87]. Overexpressed NBCe1 may be involved in the excessive sodium load and subsequent calcium overload through the sodium–calcium exchanger, which induces sodium efflux and calcium influx in cardiomyocytes [88]. Thus, sodium and calcium overload induce contractile dysfunction. Modulation of cardiac NBCe1 could be a potential strategy against cardiac contractile dysfunction and cardiac sudden death. 

In addition, drugs that modulate calcium signaling play a role in the regulation of bicarbonate transporters. Treatment with the calcium ionophore 4Br-A23187 increases intracellular calcium, while it inhibits DRA activity in DRA-transfected human embryonic kidney (HEK) cells [89]. 

Regarding calcium signaling-associated proteins, as a calcium-associated protein, IRBIT (IP_3_R coupling protein released with inositol 1,4,5-trisphosphate) has been identified as a molecule that competes with IP_3_ for binding to the IP_3_ receptor [90,91] and is now recognized as a multifactorial regulator due to its wide range of target proteins such as NBCe1-B [92] and calcium/calmodulin kinase IIα [93]. IRBIT binds to NBCe1-B, one variant of NBCe1, and regulates NBC activity, playing a critical role in epithelial secretion [92,94]. The activity of NBCe1-B exhibits basal properties due to the auto-inhibitory domain (AID), which consists of residues 1-85 [95,96]. The AID of NBCe1-B is released by the binding of IRBIT through electrostatic interaction, resulting in the activation of NBCe1-B [96,97,98]. In addition, AE2 is identified as one of the binding targets of both IRBIT and long (L)-IRBIT in HEK293T cells [99]. L-IRBIT KO via CRISPR/Cas9 induces downregulated AE2 activity and expression in B16F10 melanoma cells [99]. IRBIT/L-IRBIT double KO reduces AE2 expression, whereas IRBIT overexpression restores AE2 activity in IRBIT/L-IRBIT double KO B16-F10 cells [99]. In L-IRBIT KO B16F10 cells, the lysosomal degradation inhibitor bafilomycin upregulates AE2 protein expression and recovers L-IRBIT KO-induced AE2 downregulation [99]. IRBIT and L-IRBIT possess the AHCY domain, which forms multimer. The AE2 binds to IRBIT multimer, which contains homo-multimer or hetero-multimer in HEK293T cells [99]. Although only IRBIT homo-multimer is involved in the regulation of AE2 expression, the binding of IRBIT family modulates the protein stability of AE2 through the modulation of endosome/lysosome-dependent degradation pathway. 

Additionally, IRBIT antagonizes kinases such as Ste20-related proline/alanine-rich kinase (SPAK) or with-no-lysine (WNK) kinase, which destabilizes membrane expression of NBCe1-B [92]. Hwang et al. reported that IRBIT stabilizes NBCn1 expression in the plasma membrane to support cellular migration in A549 lung cancer cells [100]. Knockdown of IRBIT destabilizes plasma NBCn1 expression and reduces cellular migration, suggesting that IRBIT mediates the maintenance of a stable migratory module [100]. Although the calcium-associated protein IRBIT is related to several bicarbonate transporters such as AE2 and NBCs, identifying the regulatory relationships between IRBIT or IRBIT family and other bicarbonate transporters will be challenging in the future.

## 3. Host Defense Mechanisms

Bacterial prostatitis results from a bacterial infection in prostate glands, and is one of the inflammation to verify the host defense mechanism. Although we discussed the effect of inflammatory cytokines on bicarbonate transporters in Section 2.1, this section will discuss the bacterial-associated inflammation and its bicarbonate transporter regulation in detail. A common feature of prostatitis is an increase in pH, termed as an alkaline shift (>pH 8.0) [101]. Inflammatory cytokines, particularly TNF-α and IL-10, are elevated in prostatitis, and their presence has been detected in the urine of prostatitis patients [102]. These elevated inflammatory mediators induce an upregulation in CFTR expression [103]. In response to bacterial infection, prostatitis exhibits enhanced bicarbonate secretion involving CFTR, resulting in an elevation of pH [104]. The alkaline shift in pH, facilitated by bicarbonate secretion, serves as a defense mechanism against bacterial infection and inflammation. Enhanced pH of airway surface liquid plays a crucial role in respiratory host defense mechanisms, including mucociliary movement and anti-bacterial activity [105,106]. In addition, the infection of Helicobacter *pylori* leads to downregulated expressions and activities of duodenal CFTR and PAT-1 through transforming growth factor (TGF)-β-mediated MAPK pathway, which participates in the development of duodenal ulcers in non-tumorigenic duodenal epithelial cells from human patients [107].

Since bicarbonate has been shown to be involved in antibacterial activity, the reinforced CFTR-mediated bicarbonate secretion in prostatitis can function as a host defense mechanism [108,109,110,111]. For instance, *E. coli*-derived LPS treatment upregulates the expression of mRNA and protein for various inflammatory cytokines such as IL-1β, TNF-α, and IL-6 as well as the bicarbonate–associated CFTR and CA2 in prostate epithelial cells isolated from rats [104]. Inhibition of CA by acetazolamide or CFTR inhibition by CFTR (inh)-172 enhance bacterial activity in *E. coli*–injected prostate epithelial (PE) cells [104]. Consistently, mRNA and protein levels of CA2, CFTR, and pro-inflammatory cytokines are upregulated in *E. coli*–injected PE cells [104]. 

In IBD, decreased NaCl absorption is observed and results in IBD-associated diarrhea [112]. The absorption of NaCl involves the activity of NHE such as NHE3 and CBE such as DRA [112]. 

There are several studies related to anion exchange systems which involve chloride-dependent bicarbonate secretion and pH modulation. Mutations or dysregulation of DRA leads to diarrheal disorders including UC [113]. Free chloride from bicarbonate solution (containing 10 mM glucose, 25 mM NaHCO_3_, 115 mM Na^+^-gluconate, 1 mM Mg^2+^-gluconate, 6 mM Ca^2+^-gluconate, and K^+^-sulfate) induces alkaline intracellular pH in colonic crypts isolated from patients [113]. Additionally, DRA activity is enhanced in surface regions of distal colon crypts compared to the middle and bases [113]. However, DRA activity and mRNA expression level are downregulated, and subsequent chloride absorption is decreased in surface crypts of UC patients [112,113]. Additionally, a similar decrease in DRA mRNA expression is also observed in IL-10 KO mice after developing colonic inflammation [15]. Inflammatory diseases are attenuated via the inhibition of p-STAT signaling. For instance, all-trans retinoic acid (ATRA) treatment upregulates DRA activity and protein expression in interferon (IFN)-γ-treated Caco-2 cells [114]. Moreover, ATRA treatment downregulates IFN-γ-induced enhanced p-STAT1 expression, reducing the severity of inflammation in DSS-induced colitis mice and its isolated primary colon tissues [114].

## 4. Bicarbonate-Associated Carbonic Anhydrases

Carbonic anhydrases (CAs) are enzymes responsible for various biological processes such as cellular pH homeostasis, ion transport, cell respiration, and gluconeogenesis, and they function to catalyze the reversible reaction: CO_2_ + H_2_O ⇄ HCO_3_^−^ + H^+^ [115,116]. The human CAs consist of 15 isoforms and exist in various cellular locations: there are cytosolic isoforms (CA1, CA2, CA3, CA7, and CA8), mitochondrial isoforms (CA5-A and CA5-B), a secretory isoform (CA6), and membrane-coupled isoforms (CA4, CA9, CA12, CA14, and CA15) [116,117,118,119,120,121]. CAs are an enzymatic source of bicarbonate and thus are closely related to the activity of bicarbonate transporters. CA2, for example, induces an upregulation of NBCe1 activity in *Xenopus* oocytes as determined by elevations in intracellular sodium concentration, and increases membrane currents and conductance [122]. The membrane current is downregulated via treatment with CA inhibitor, ethoxyzolamide, in CA1-NBCe1 and CA3-NBCe1 co-expressing oocytes [123]. Furthermore, CA1 protein injection increases NBCe1 activity and its catalytic activity in oocytes [123]. 

CA4 is a membrane-attached enzyme with a glycosyl-phosphatidyl-inositol anchor and is involved in bicarbonate transport in brain, lung, kidney, and muscle [118,124]. In particular, CA4 interacts with bicarbonate transporters, such as AE2 and NBCe1, to regulate intracellular pH [125]. Mechanically, CA4 catalyzes the reversible reaction to produce carbon dioxide. The carbon dioxide diffuses into the cells, or bicarbonate serves as a source of bicarbonate influx mediated by NBCe1-B, thereby participating in the bicarbonate metabolon that regulates intracellular pH [125]. The CA4 protein is widely expressed in hypoxic tumors and CA4 overexpression upregulates cell migration in Madin–Darby canine kidney (MDCK) cells with or without hepatocyte growth factor (HGF), an epithelial-mesenchymal transition inducer [126]. In addition, hypoxia induces co-localization of CA4 with AE2 and NBCe1 at the leading-edge regions of migrating SiHa and A549 cells [126], suggesting that bicarbonate transporters provide mechanical machinery for movement of cells. 

CA12 is a plasma membrane-associated enzyme which can be described as an oncogenic marker such as CA9 [127,128,129]. CA12 co-localizes with NBCe1 in the cellular membrane of MDCK and HEK cells [130]. However, a mutation in CA12 (E143K) results in mis-localization of the protein to the ER in transfected MDCK or HEK cells [130]. The mutated CA12 (E143K) downregulates the activities of NBCe1 and AE2 in HeLa cells [130]. This CA12 mutation is found in patients presenting with symptoms of dry mouth and dry tongues which occur due to reduced fluid secretion [130], suggesting that downregulation of NBCe1 and AE2 via CA12 mutation are involved in reduced fluid secretion and salivation. We summarized the biological effect of CAs on the modulation of bicarbonate transporters (Table 1).

It also has been found that the conversion ratio of bicarbonate to carbon dioxide regulates the formation of neutrophil extracellular trap around inflammation foci [131]. Involvement of CA activity and bicarbonate transporter activity with conversion ability may regulate the conversion ratio through the modulation of bicarbonate content. Thus, modulation of CA or bicarbonate transporter activity on a neutrophil extracellular trap might be considered as potential strategies to attenuate inflammation foci.

## 5. Acidic Microenvironment and Proliferation

Hypoxic condition and production of acidic metabolites such as lactate, carbon dioxide, and proton ion are essential processes of cancer progression [5,132]. It has been known that progression of breast cancer, lung cancer, pancreatic cancer, and melanoma is associated with acidic stress and the microenvironment [100,133,134,135,136]. Although alkaline-pH-shift mechanism in urine against bacterial infection should be distinguished, acidic stress stimulates cancer cell survival and migration through the involvement of bicarbonate transporters such as NDCBE and NBCn1 [5]. NDCBE expression was also enhanced by acid loads in the rat brain [100,137]. In addition, an acidic environment in the inflamed synovium in rheumatoid arthritis (RA) triggers membrane recruitment of NBCn1 in fibroblast-like synoviocytes (FLS), mediating the aggressiveness of RA-FLS [138]. More recently, the stimulation of insulin and growth factor enhances NBCn1 protein expression in HeLa cells [139]. The mechanistic target of rapamycin complex 1 (mTORC1)-S6K1 axis stimulates the transcription of SLC4A7 mRNA [139]. Depleted *Slc4a7*-mice reveal reduced tumor growth in CAL-51 breast cancer cell line-implanted xenograft mice and thus recruited NBCn1 protein into the plasma membrane, and subsequent enhancement of bicarbonate influx is involved in de novo nucleotide synthesis and the cell growth of the tumor [139]. Although it has been found that mTORC1 signaling modulates NBCn1 expression in proliferating cells, future studies on the mechanism remain to be carried out for other pathophysiological states of cells and specific tissues. 

## 6. Conclusions and Perspectives

Regulatory signals or proteins that act on the modulation of bicarbonate transporters are discussed in physiological and pathological conditions (Table 2). Inflammatory cytokines, peptides, and enzymes, mentioned in this review, are involved in the modulation of bicarbonate flux through specific bicarbonate transporters, thereby regulating cellular pH and various pathophysiological roles. Although not all members of the bicarbonate transporter family are discussed here, the large number of known isotypes provide potential research themes that need investigating in physiological or pathological conditions. In addition, although structural homology of bicarbonate transporters must be overcome, development of a specific activator or inhibitor on bicarbonate transporters will be an attractive research area. Thus, we are challenged to gain greater understanding of activating mechanisms and to identify new activators and inhibitors to modulate specific bicarbonate transporters. Thus, future additional verification of mechanisms of the modulation of bicarbonate transporters will be beneficial to obtain new paradigms in bicarbonate transporter-associated whole-body systems and diseases, such as immune, cancer, respiratory, tooth development, digestive, cardiac, and reproductive systems.

## Figures and Tables

**Table 1:** Biological effects of CAs on bicarbonate transporters.

Isoform Type	Transporters	Related Mechanisms	Species	Ref
CA1 (Microinjection)	NBCe1	Increased NBC activity and catalytic activity	Oocytes	[123]
CA2 (Microinjection)	NBCe1	Elevated NBC activity	Oocytes	[122]
CA2 (Transfection)	AE2 NBCe1	Upregulated AE2 activities No effect on NBC activity	HeLa cells	[130]
CA4 (Transfection)	AE2, NBCe1	Regulation of intracellular pH	HEK293T	[125]
CA12 (Transfection)	NBCe1, AE2	Upregulated NBC and AE2 activities	HeLa cells	[130]
CA12 (E143K) (Transfection)	NBCe1, AE2	Downregulated NBC and AE2 activities, reduced fluid secretion	HeLa and primary parotid gland cells	

**Table 2:** Various stimulants of bicarbonate transporters.

Type	BT	Ions	Expression Tissues	Related Diseases	Stimulants	Ref
CBE; SLC26A family	DRA (SLC26A3)	Cl−, HCO_3_^−^	Colon, ileal, and intestine	Bowel diseases, Inflammatory	Inflammatory cytokines (IL-17 and TNF-α), Caffeic acid phenethyl ester, NPY, MβCD, 4Br-A23187, and ATRA	[19,21,25,79,89,114]
	Pendrin (SLC26A4)		Respiratory tract, kidney	Inflammatory, Dysregulation of airway surface liquid pH	TNF-α, Ang II, IL-17	[14,25,46]
	PAT-1		Duodenal	*H. pylori*-associated duodenal ulcer	*H. pylori*	[107]
CBE; Anion exchanger family	AE1	Cl^−^, HCO_3_^−^	Kidney	Maintenance of electrolyte and acid-base disturbances	Acidic stress, alkaloid stress	[45]
	AE2		Skin, kidney, and liver	Melanoma, Primary biliary cholangitis.	L-IRBIT K/O, IRBIT/L-IRBIT double K/O, and pro-inflammatory cytokines (IL-8, 12, 17, 18, and TNF-α)	[23,99]
	AE3		Heart	Cardiac hypertrophy	Ang II	[44]
NBC	NBCe1	Na^+^, HCO_3_^−^	Heart, airway, pancreatic ducts, parotid ducts, and oocytes	Hypertrophy, heart failure	IL-17 + TNF-α, ANG II, IRBIT, CA1, and CA2	[14,41,92,122,123]
	NBCn1		Airway, heart, lung, and cervix	Hypertrophy, heart failure, carcinoma, adenocarcinoma	IL-17 + TNF- α, ANG II, IRBIT, Insulin, and growth factor	[14,41,100,139]
	NDCBE	Cl^−^, HCO_3_^−^	Brain, skin	Chronic metabolic acidosis, melanoma	Acidic stress	[5,137]
ATP-binding cassette transporter	CFTR	Cl^−^, HCO_3_^−^	Duodenal, airway, brain, lung, and prostate	*H. pylori*-associated duodenal ulcer, hypertension, adenocarcinoma	*H. pylori*, TNF-a + IL17, ANG II, VIP, LPS, *E. coli*	[14,47,59,104,107]

Abbreviations: CBE, chloride–bicarbonate exchanger; SLC26A, solute carrier family 26; DRA, downregulated in adenoma; PAT-1, putative anion transporter 1; IL, interleukin; TNF-α, tumor necrosis factor α; NPY, neuropeptide Y; MβCD, methyl-β-cyclodextrin; ATRA, all-trans retinoic acid; Ang II, angiotensin II; AE, anion exchanger; L-IRBIT, long-IP_3_R coupling protein released with inositol 1,4,5-trisphosphate; IRBIT, IP_3_R coupling protein released with inositol 1,4,5-trisphosphate; NBC, sodium-bicarbonate cotransporter; NBCe1, electrogenic sodium bicarbonate cotransporter1; NBCn1, electroneutral sodium bicarbonate cotransporter1; NDCBE, sodium-dependent chloride-bicarbonate exchanger; CA, carbonic anhydrase; CFTR, cystic fibrosis transmembrane conductance; VIP, vasoactive intestinal peptide; LPS, lipopolysaccharide; *H. pylori*, *Helicobacter pylori*; *E. coli*, *Escherichia coli*.
